# Decreased high density lipoprotein cholesterol is an independent predictor for persistent organ failure, pancreatic necrosis and mortality in acute pancreatitis

**DOI:** 10.1038/s41598-017-06618-w

**Published:** 2017-08-14

**Authors:** Yushun Zhang, Feng Guo, Shoukang Li, Feiyang Wang, Zibo Meng, Jingyuan Zhao, Zhiqiang Liu, Bo Wang, Ping Fan, Chunyou Wang, Heshui Wu

**Affiliations:** Department of Pancreatic Surgery, Union Hospital, Tongji Medical College, Huazhong University of Science and Technology, Wuhan 430022, China

## Abstract

High density lipoprotein cholesterol (HDL-C) has been reported as a significant indicator of systemic inflammation. The association underlying HDL-C and persistent organ failure (POF), pancreatic necrosis (PNec) and mortality in acute pancreatitis (AP) has not been evaluated. From 2007 to 2016, consecutive AP patients with admission lipid profiles assessment were included in this study. The association of HDL-C value and other lipids with outcomes was explored with Cox proportional regression models, which were adjusted for confounding factors. 1131 consecutive AP patients were clinically eligible. Overall, 17.9% of the patients developed with POF, 27.1% experienced PNec, and 6.7% died during hospitalization. Lower HDL-C median (<1.06 mmol/L) was identified as an independent prognostic factor of the outcomes. Moreover, there was a positive trend for the association across increasing HDL-C quartiles and POF, PNec and mortality after multivariable analysis (p values were <0.001, <0.001 and 0.043, respectively). The AUC of HDL-C for the outcomes were comparable to that of Ranson score for diagnosing POF (0.778 vs. 0.678; P < 0.001), PNec (0.734 vs. 0.701; P = 0.143) and mortality (0.768 vs. 0.745; P = 0.516). Decreased HDL-C value is an independent risk factor for the incidence of POF, PNec and in-hospital mortality in AP.

## Introduction

Acute pancreatitis (AP) is characterized by local and systemic inflammation, which is observed clinically from no systemic sign through the local and systemic inflammatory response, organ failure (OF), persistent organ failure (POF), pancreatic necrosis (PNec) and death^1, 2^. The underlying pathophysiology through which local pancreatic injury drives the systemic inflammatory response has not been fully elucidated, but cumulative data suggest that immune systems play pivotal roles^3, 4^. Impaired lipid metabolism plays a pivotal role in the pathogenesis of numerous diseases conditions, including cardiovascular conditions, infectious diseases diabetes and carcinoma^5–9^.

Compared with other lipoproteins, high density lipoprotein cholesterol (HDL-C) is carried within lipoprotein particles that are particularly heterogeneous, varying in size, composition, metabolism and function^10^. Decreased plasma concentrations of HDL-C is frequently observed in a serious of acute phase conditions associated with immune activation^11^. It is accepted that HDL may become dysfunctional in some disease conditions, and the concept that the anti-inflammatory status of HDL may be associated with the risk of cardiovascular and other disorders^12–14^. In addition to playing a central role in reverse cholesterol transport (RCT) in vascular protection, HDL and its mimetics have several other functions including antioxidant, antithrombotic and anti-apoptotic functions. The anti-inflammatory function of HDL is investigated intensively, affecting both local (such as expression of adhesion molecules on endothelium) and systemic immune systems (such as expression of adhesion molecules and cytokine secretion by monocytes)^15^.

HDL-C has been shown an association with disease severity and adverse outcomes in AP^16–21^. However, evidence supporting the relationship between HDL-C and incidence of POF, PNec and in-hospital mortality in a large population of AP, is currently lacking. In the present study, we aimed to evaluate whether there is an association between HDL-C and severe outcomes in patients with AP.

## Materials and Methods

### Study Population

A total of 3004 consecutive patients who admitted to the Pancreatic Disease Institute of Union Hospital (Wuhan, China) with a confirmed diagnosis of AP between January 2007 and January 2016 were included in this retrospective study. AP was defined as clinical findings based on the presence of two or more of the following three criteria: (1) abdominal pain consistent with AP; (2) serum amylase and/or lipase elevation ≥ three times the upper limit of normal; and/or (3) computed tomography (CT), magnetic resonance imaging (MRI) or abdominal ultrasonography findings characteristic of AP^1^. Of these, 1532 patients were admitted to hospital within 72 hours from symptom onset and underwent assessment of lipid profiles upon presentation. For this study, individuals were excluded if they met any of the following: age smaller than 18 years (n = 107), AP induced by trauma (n = 78), chronic pancreatitis (n = 223), a history of hyperlipidemia receiving statin treatment currently (n = 171), and unavailable laboratory measurements or medical records (n = 346). As some individuals met more than one exclusion criteria, the total number of eligible patients for the study was 1131 (Supplementary Figure 1). The study protocol conformed to the ethical guidelines of the 1975 Declaration of Helsinki. The local Ethics Committee of Union Hospital (Wuhan, China) approved this study. Informed consent was obtained from each patient or next of kin prior to the study.

### Data Collection

Electronic medical records and paper charts were reviewed by one independent physician who was blinded to the study for information on demographics (age, sex, etiology), medical history, smoking habit, alcohol consumption, pre-existing co-morbidity such as diabetes mellitus, obesity, and pre-existing organ dysfunction (including chronic respiratory disease, chronic renal disease and cardiovascular disease). Blood samples were collected within 2 hours after hospitalization and analyzed using an automated clinical chemical analyzer within 6 hours of sampling in the same core clinical laboratory in Union Hospital (Wuhan, China). Whole blood samples were obtained for measurements of hemoglobin, and serum for creatinine, calcium, albumin, aspartate aminostransferase, triglyceride (TG), total cholesterol (T-CHO), LDL-C, and HDL-C levels. The values of TG, T-CHO, HDL-C and LDL-C were measured enzymatically. TG/HDL-C ratio was calculated as TG level (mmol/L) divided by HDL-C level (mmol/L). Non-HDL-C was defined as T-CHO (mmol/L) minus HDL-C level (mmol/L). Because the TG, T-CHO, TG/HDL-C ratio and non-HDL-C were not normally distributed in the AP patients, we constructed plots of them and HRs of the outcomes using the Lowess function. The results revealed a nonlinear relationship, suggesting the need for stratification of the patients into medians and quartiles according to their values for outcome analysis. Therefore, we stratified the patients into medians and quartiles according to their TG, T-CHO, HDL-C level, LDL-C level, TG/HDL-C ratio or non-HDL-C level. The primary predictor variable was the HDL-C in each quartile. We also selected TG, T-CHO, LDL-C, TG/HDL-C ratio and non-HDL-C as other predictors.

### Outcomes

The outcomes were incidence of developing with POF, PNec and in-hospital mortality. OF was confirmed according to the 2012 Revised Atlanta criteria^1^ when the following cutoffs were exceeded: (1) cardiovascular failure if systolic blood pressure was <90 mmHg despite fluid replacement; (2) respiratory failure if the ratio of PaO_2_/FiO_2_ was <300 mmHg; and (3) renal failure if serum creatinine was ≥1.9 mg/dl. Presence of OF was assessed upon presentation and every 24 hours during hospitalization. POF was identified if OF lasting more than 48 hours. PNec was defined as appearance of pancreatic parenchymal and/or peri-pancreatic necrosis on contrast-enhanced computed tomography (CECT)^1^. In our department, patients will receive CT and/or MRI on admission to determine the presence of pancreatitis. As pancreatic and peri-pancreatic necrosis usually is not present upon presentation and may develop during the first few days, early imaging cannot reliably determine severity in the course of AP. A CECT 7–9 days after admission will be repeated to establish the presence and extent of PNec. The outcome information was centrally adjudicated, in accordance with above definitions, by trained clinicians and experienced radiologists who were blind to this study.

### Statistical Analysis

Statistical analysis was performed using SPSS 20.0 (SPSS Inc, Chicago IL, USA). Continuous data are presented as mean and standard deviation (SD). Categorical data are reported as number (frequency). Student *t* test and Mann-Whitney *U* test were used to evaluate the differences of baseline characteristics between the study cohort and the control group. Multiple group comparisons were performed using the Chi-square test for categorical variables and the Kruskal-Wallis test for continuous data. Univariable, age and sex adjusted, or multivariable analyses for outcomes were performed using a logistic regression model. The multivariable covariates included age, sex, smoking habit, alcohol use, pre-existing comorbidities and admission laboratory data. Hazard ratios (HRs) and 95% confidence intervals (95% CIs) are presented. P values were 2-sided, and a P value < 0.05 was considered to be statistically significant. For testing linear risk trends, we used the median and quartile rank as a continuous variable in the regression models. We checked the proportional HRs by examining graphs of estimated log (−log) survival. To further evaluate and compare the predictive performance of TG, T-CHO, HDL-C, LDL-C, TG/HDL-C ratio and non-HDL-C, we used receiver operating characteristic (ROC) curves, and the area under the ROC curve (AUC) was estimated. The predictive values were compared with conventional prognostic system of Ranson score.

### Data availability statement

We declare that the data is available.

## Results

### Basic Characteristics and outcomes of the patients

Baseline characteristics of the AP patients are presented in Table 1. The mean age of the population was 46 years and 683 (60.4%) were males. The most common cause was biliary-induced AP (n = 565), followed by alcoholic-induced AP (n = 282) and hyperlipidemia-induced AP (n = 196), and 7.8% with other causes. The mean values of TG, T-CHO, HDL-C, LDL-C, TG/HDL-C and non-HDL-C upon admission for the entire population were 4.91 ± 8.49 mmol/L, 4.99 ± 3.01 mmol/L, 1.07 ± 0.40 mmol/L, 1.91 ± 0.89 mmol/L, 7.46 ± 18.54, and 3.92 ± 3.09 mmol/L, respectively (Table 1).

**Table 1:** Baseline characteristics of the study patients with acute pancreatitis.

	All patients
No.	1131
Age, years	46.42 ± 14.87
Male sex	683 (60.4%)
Etiology	
Biliary	565 (50.0%)
Alcoholic	282 (24.9%)
Hyperlipidemia	196 (17.3%)
Other cause	88 (7.8%)
Lipid files	
TG, mmol/L	4.91 ± 8.49
TC, mmol/L	4.99 ± 3.01
HDL-C, mmol/L	1.07 ± 0.40
LDL-C, mmol/L	1.91 ± 0.89
TG/HDL-C	7.46 ± 18.54
Non-HDL-C, mmol/L	3.92 ± 3.09
Outcomes	
POF	203 (17.9%)
Solitary POF	119
Respiratory	119
Renal	0
Cardiovascular	0
Multiple POF	84
Respiratory + renal	65
Respiratory + cardiovascular	12
Respiratory + cardiovascular + renal	7
PNec	306 (27.1%)
<30%	55
30–50%	128
>50%	123
In-hospital mortality	76 (6.7%)

Data are presented as mean (SD) and number (frequency).

Abbreviations: CI: confident interval; HDL-C, high-density lipoprotein cholesterol; HR, hazard ratio; LDL-C, low-density lipoprotein cholesterol; PNec, pancreatic necrosis; POF, persistent organ failure; T-CHO, total cholesterol; TG, triglycerides.

Overall, 203 (17.9%) of the patients were diagnosed with POF. 119 patients had solitary POF (all in respiratory system). Multiple POF was observed in 84 patients (65 of lung and kidney, 12 of lung and heart, and 7 of lung, kidney and heart). Accordingly, 306 (27.1%) patients developed PNec. During hospitalization, 76 patients with POF died with an overall mortality rate of 6.7%. No death was observed in non-POF ones. Accordingly, 21 patients died from persistent respiratory failure, 17 from persistent respiratory and renal failure, 13 from persistent respiratory and cardiac failure, and 25 from infected PNec.

**Figure 1:**
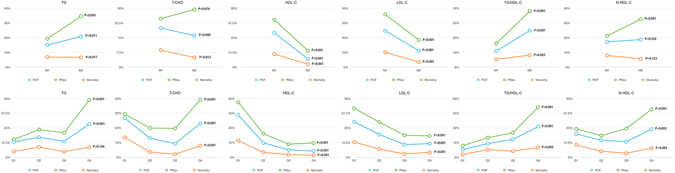
Comparison of the incidences of POF, PNec and in-hospital mortality in patients with AP according to medians and quartiles of different lipid profiles. Abbreviations: AP, acute pancreatitis; HDL-C, high-density lipoprotein cholesterol; HR, hazard ratio; LDL-C, low-density lipoprotein cholesterol; PNec, pancreatic necrosis; POF, persistent organ failure; T-CHO, total cholesterol; TG, triglycerides.

When these patients were stratified according to medians of these lipid profiles, the incidence of POF was more prevalent in patients with higher levels of TG and TG/HDL-C, and lower levels of HDL-C and LDL-C. The incidence of PNec was statistically elevated in patients with higher levels of TG, TG/HDL-C and non-HDL-C, and lower levels of HDL-C and LDL-C. Mortality rate was significantly higher in patients with lower levels of T-CHO, HDL-C and LDL-C (Fig. 1). Meanwhile, when patients were stratified according to quartiles of various lipid parameters, the incidences of POF, PNec and in-hospital mortality remained significantly different among quartiles (Fig. 1).

### Comparison of clinical parameters and lipid profiles between patients with different outcomes

Table 2 shows that males and patients with drinking and smoking habits were more prone to develop PNec. POF ones and non-survivors showed elder ages. The values of total leukocytes, aspartate aminostransferase, serum glucose, creatinine, TG, TG/HDL-C, non-HDL-C detected upon presentation to hospital and Ranson score were significantly higher, while the admission levels of serum calcium, HDL-C and LDL-C were statistically lower in patients with severe outcomes (Table 2).

**Table 2:** Comparison of baseline characteristics and outcomes between patients with acute pancreatitis.

	Non-POF	POF	P	Non-PNec	PNec	P	Survior	Non-survior	P
	928	203		825	306		1055	76	
Male sex	563 (60.7%)	120 (59.1%)	0.682	473 (57.3%)	210 (68.6%)	<0.001	643 (60.9%)	40 (52.6%)	0.152
Age, years	44.02 ± 14.16	51.51 ± 16.06	<0.001	44.91 ± 14.83	46.59 ± 14.65	0.212	44.57 ± 14.30	56.39 ± 17.08	<0.001
Etiology			<0.001			<0.001			0.077
Biliary	461 (49.7%)	104 (51.2%)		442 (53.6%)	123 (40.2%)		519 (49.2%)	46 (60.5%)	
Alcoholic	216 (23.3%)	66 (32.5%)		147 (17.8%)	135 (44.1%)		262 (24.8%)	20 (26.3%)	
Hyperlipidemia	167 (18.0%)	29 (14.3%)		158 (19.2%)	38 (12.4%)		188 (17.8%)	8 (10.5%)	
Other cause	84 (9.1%)	4 (2.0%)		78 (9.5%)	10 (3.3%)		86 (8.2%)	2 (2.6%)	
Daily drinker	416 (44.8%)	96 (47.3%)	0.523	333 (40.4%)	179 (58.5%)	<0.001	482 (45.7%)	30 (39.5%)	0.239
Current smoker	468 (50.4%)	100 (49.3%)	0.763	376 (45.6%)	192 (62.7%)	<0.001	540 (51.2%)	28 (36.8%)	0.016
Diabetes mellitus	85 (9.2%)	77 (37.9%)	<0.001	85 (10.3%)	77 (25.2%)	<0.001	126 (11.9%)	36 (47.4%)	<0.001
Obesity	146 (15.8%)	46 (22.7%)	<0.001	122 (14.8%)	70 (22.9%)	0.001	180 (17.1%)	12 (15.8%)	0.773
Pre-existing organ dysfunctions	218 (23.5%)	91 (44.8%)	0.118	215 (26.1%)	94 (30.7%)	0.102	265 (25.1%)	44 (57.9%)	<0.001
Chronic respiratory disease	195 (21.0%)	78 (38.4%)	0.247	199 (24.1%)	74 (24.2%)	0.978	229 (21.7%)	44 (57.9%)	<0.001
Chronic renal disease	9 (1.0%)	16 (7.9%)	<0.001	3 (0.4%)	22 (2.7%)	<0.001	2 (0.2%)	23 (30.3%)	<0.001
Cardiovascular disease	34 (3.7%)	36 (17.7%)	<0.001	28 (3.4%)	42 (13.7%)	<0.001	32 (3.0%)	38 (50.0%)	<0.001
Laboratory data									
Total leukocyte count, ×10^9^/L	11.28 ± 4.23	14.49 ± 6.42	<0.001	11.10 ± 4.21	13.90 ± 5.80	<0.001	11.71 ± 4.80	13.93 ± 5.14	<0.001
Aspartate aminostransferase, U/L	73.38 ± 118.23	92.59 ± 102.17	<0.001	73.65 ± 119.48	85.40 ± 104.55	<0.001	73.98 ± 115.80	116.34 ± 107.41	<0.001
Serum glucose, mmol/L	7.23 ± 2.65	11.53 ± 6.67	<0.001	7.01 ± 2.36	10.68 ± 6.01	<0.001	7.59 ± 3.07	13.72 ± 8.98	<0.001
Serum calcium, mmol/L	2.14 ± 0.24	1.71 ± 0.37	<0.001	2.16 ± 0.22	1.81 ± 0.38	<0.001	2.10 ± 0.27	1.60 ± 0.40	<0.001
Creatinine, mmol/L	64.94 ± 21.07	143.35 ± 125.64	<0.001	64.00 ± 19.55	119.51 ± 108.96	<0.001	70.04 ± 36.36	203.64 ± 161.87	<0.001
Ranson score	3.32 ± 1.82	5.00 ± 1.83	<0.001	3.13 ± 1.71	4.95 ± 1.87	<0.001	3.50 ± 1.90	5.34 ± 1.49	<0.001
TG, mmol/L	4.14 ± 6.85	8.44 ± 13.14	<0.001	3.52 ± 6.25	8.65 ± 11.93	<0.001	4.77 ± 8.14	6.86 ± 12.31	0.038
T-CHO, mmol/L	4.81 ± 2.44	5.82 ± 4.76	<0.001	4.62 ± 2.31	5.99 ± 4.22	<0.001	4.96 ± 2.82	5.36 ± 4.99	0.264
HDL-C, mmol/L	1.14 ± 0.37	0.75 ± 0.39	<0.001	1.16 ± 0.38	0.83 ± 0.40	<0.001	1.04 ± 0.43	0.70 ± 0.39	<0.001
LDL-C, mmol/L	1.99 ± 0.87	1.54 ± 0.90	<0.001	2.03 ± 0.84	1.59 ± 0.94	<0.001	1.94 ± 0.87	1.48 ± 1.03	0.068
TG/HDL-C	6.42 ± 14.50	18.64 ± 35.00	<0.001	5.59 ± 13.77	16.66 ± 30.30	<0.001	7.70 ± 17.64	20.47 ± 40.50	<0.001
Non-HDL-C, mmol/L	3.75 ± 2.49	5.09 ± 4.83	<0.001	3.55 ± 2.34	5.17 ± 4.29	<0.001	3.93 ± 2.86	4.74 ± 5.14	0.027

Data are presented as mean (SD) and number (frequency).

Abbreviations: CI: confident interval; HDL-C, high-density lipoprotein cholesterol; HR, hazard ratio; LDL-C, low-density lipoprotein cholesterol; PNec, pancreatic necrosis; POF, persistent organ failure; T-CHO, total cholesterol; TG, triglycerides.

### Association between different lipid profiles and the three outcomes

1. HDL-C and LDL-C are independently associated with outcomes
  We next evaluated the association of various lipid parameters at presentation with the three AP outcomes. Table 3 shows HRs and 95% CIs for POF, PNec and in-hospital mortality using lipid profiles as continuous variables. In univariate analysis, lipid files such as TG, HDL-C, LDL-C, TG/HDL-C and non-HDL-C were significantly associated with the three outcomes.

**Table 3:** Factors associated with outcomes using univariate and multivariate Cox proportional analyses.

	POF	*P*-value	PNec	*P*-value	Mortality	*P*-value
	HR (95%CI)		HR (95%CI)		HR (95%CI)	
Univariate						
Female sex (1 = yes, 0 = no)	1.07 (0.78, 1.45)	0.682	0.61 (0.47, 0.81)	**<**0.001	1.40 (0.88, 2.24)	0.153
Age (years)	1.04 (1.02, 1.05)	**<**0.001	1.01 (1.00, 1.02)	0.090	1.06 (1.04, 1.07)	**<**0.001
Biliary etiology (1 = yes, 0 = no)	1.06 (0.79, 1.44)	0.688	0.58 (0.45, 0.76)	**<**0.001	1.58 (0.98, 2.25)	0.058
Daily drinker (1 = yes, 0 = no)	1.10 (0.81, 1.50)	0523	2.08 (1.60, 2.72)	**<**0.001	0.78 (0.48, 1.25)	0.294
Current smoker (1 = yes, 0 = no)	0.95 (0.70, 1.29)	0.763	2.01 (1.54, 2.63)	**<**0.001	0.56 (0.34, 0.90)	0.017
Diabetes mellitus (1 = yes, 0 = no)	6.06 (4.23, 8.69)	**<**0.001	2.93 (2.08, 4.12)	**<**0.001	6.64 (4.08, 10.80)	**<**0.001
Obesity (1 = yes, 0 = no)	1.57 (1.08, 2.28)	0.018	1.71 (1.23, 2.37)	0.001	0.91 (0.48, 1.72)	0.773
Pre-existing organ dysfunctions (1 = yes, 0 = no)	2.65 (1.93, 3.63)	**<**0.001	1.26 (0.94, 1.68)	0.119	4.10 (2.55, 6.60)	**<**0.001
Total leukocyte count (×10^9^/L)	1.10 (1.07, 1.14)	**<**0.001	1.13 (1.10, 1.17)	**<**0.001	1.08 (1.03, 1.12)	**<**0.001
Aspartate aminostransferase (U/L)	1.00 (1.00, 1.00)	0.034	1.00 (1.00, 1.00)	0.131	1.00 (1.00, 1.00)	0.003
Serum glucose (mmol/L)	1.26 (1.21, 1.32)	**<**0.001	1.32 (1.26, 1.39)	**<**0.001	1.24 (1.18, 1.30)	**<**0.001
Serum calcium (mmol/L)	0.01 (0.00, 0.02)	**<**0.001	0.01 (0.01, 0.02)	**<**0.001	0.02 (0.01, 0.05)	**<**0.001
Creatinine (mmol/L)	1.03 (1.03, 1.04)	**<**0.001	1.03 (1.02, 1.03)	**<**0.001	1.03 (1.02, 1.03)	**<**0.001
Ranson score	1.60 (1.47, 1.76)	**<**0.001	1.77 (1.62, 1.94)	**<**0.001	1.59 (1.41, 1.79)	**<**0.001
TG (mmol/L)	1.05 (1.03, 1.06)	**<**0.001	1.07 (1.05, 1.09)	**<**0.001	1.02 (1.00, 1.04)	0.042
T-CHO (mmol/L)	1.09 (1.05, 1.14)	**<**0.001	1.15 (1.10, 1.20)	**<**0.001	1.04 (0.97, 1.11)	0.266
HDL-C (mmol/L)	0.16 (0.10, 0.23)	**<**0.001	0.25 (0.18, 0.34)	**<**0.001	0.14 (0.08, 0.26)	**<**0.001
LDL-C (mmol/L)	0.51 (0.42, 0.63)	**<**0.001	0.53 (0.45, 0.63)	**<**0.001	0.50 (0.36, 0.69)	**<**0.001
TG/HDL-C	1.02 (1.02, 1.03)	**<**0.001	1.03 (1.02, 1.04)	**<**0.001	1.02 (1.01, 1.02)	**<**0.001
Non-HDL-C (mmol/L)	1.12 (1.07, 1.17)	**<**0.001	1.17 (1.12, 1.23)	**<**0.001	1.07 (1.01, 1.13)	0.030
Multivariate						
Female sex (1 = yes, 0 = no)	1.15 (0.54, 2.42)	0.721	1.50 (0.79, 2.87)	0.218	1.11 (0.42, 2.30)	0.836
Age (years)	1.03 (1.01, 1.06)	0.003	1.01 (0.99, 1.02)	0.506	1.05 (1.02, 1.09)	0.003
Biliary etiology (1 = yes, 0 = no)	1.37 (0.82, 2.29)	0.227	0.95 (0.63, 1.44)	0.815	1.18 (0.53, 2.61)	0.686
Daily drinker (1 = yes, 0 = no)	2.139 (1.16, 4.95)	0.018	1.51 (0.86, 2.67)	0.151	3.61 (1.18, 11.07)	0.023
Current smoker (1 = yes, 0 = no)	0.51 (0.25, 1.04)	0.064	1.79 (0.96, 3.34)	0.066	0.22 (0.08, 0.60)	0.003
Diabetes mellitus (1 = yes, 0 = no)	3.74 (2.29, 6.11)	**<**0.001	2.54 (1.62, 3.09)	**<**0.001	2.19 (1.14, 4.22)	0.018
Obesity (1 = yes, 0 = no)	0.98 (0.56, 1.72)	0.940	0.94 (1.23, 2.37)	0.001	0.67 (0.27, 1.67)	0.386
Pre-existing organ dysfunctions (1 = yes, 0 = no)	1.34 (0.70, 2.57)	0.378	1.07 (0.60, 1.91)	0.817	1.25 (0.46, 3.39)	0.663
Ranson score*	1.23 (1.09, 1.38)	**<**0.001	1.40 (1.27, 1.55)	**<**0.001	1.35 (1.13, 1.61)	**<**0.001
TG (mmol/L)	1.02 (0.97, 1.08)	0.365	1.02 (0.98, 1.08)	0.333	0.90 (0.83, 0.99)	0.032
T-CHO (mmol/L)	1.07 (0.96, 1.17)	0.183	1.06 (0.96, 1.17)	0.249	1.11 (0.99, 1.25)	0.075
HDL-C (mmol/L)	0.09 (0.05, 0.17)	**<**0.001	0.22 (0.13, 0.36)	**<**0.001	0.13 (0.05, 0.32)	**<**0.001
LDL-C (mmol/L)	0.75 (0.57, 0.98)	0.037	0.73 (0.59, 0.92)	0.006	0.70 (0.46, 1.06)	0.091
TG/HDL-C	1.00 (0.99, 1.02)	0.756	1.00 (0.98, 1.02)	0.883	1.04 (1.01, 1.07)	0.006
Non-HDL-C (mmol/L)**	—		—		—	

*As data regarding total leukocyte count, aspartate aminostransferase, glucose, calcium and creatinine are parts of Ranson scoring system, we decide to only include Ranson score in multivariate analysis.

**Since non-HDL-C was defined as T-CHO minus HDL-C level, we decide to exclude it in multivariate analysis.

Abbreviations: CI: confident interval; HDL-C, high-density lipoprotein cholesterol; HR, hazard ratio; LDL-C, low-density lipoprotein cholesterol; PNec, pancreatic necrosis; POF, persistent organ failure; T-CHO, total cholesterol; TG, triglycerides.
  After multivariate adjustments for confounding factors, HDL-C and LDL-C remained independent prognostic factors for POF (HR 0.09, 95% CIs 0.05 to 0.17; P < 0.001 and HR 0.75, 95% CIs 0.57 to 0.98; P = 0.037) and PNec (HR 0.22, 95% CIs 0.13 to 0.36; P < 0.001 and HR 0.73, 95% CIs 0.59 to 0.92; P = 0.006). Meanwhile, HDL-C was statistically associated with in-hospital mortality (HR 0.13, 95%CIs 0.05 to 0.32; P < 0.001).
2. Higher HDL-C median is independently correlated with outcomes
  Table 4 shows HRs and 95% CIs for outcomes by medians of lipid values with median 1 (lower median) as reference. In unadjusted and age- and sex- adjusted analyses, higher medians of TG, HDL-C, LDL-C and TG/HDL-C were significantly associated with POF, and higher medians of lipid parameters (except for T-CHO) were statistically correlated with PNec. Higher medians of TG, T-CHO, HDL-C, LDL-C and TG/HDL-C were significantly associated with mortality in unadjusted analysis. After adjustments for sex and age, these lipid profiles (except for T-CHO) remained consistent as statistically predictive factors. In multivariate-adjusted analysis, results suggested higher median of HDL-C was independently related to POF (HR 0.28, 95% CIs 0.17 to 0.45; P < 0.001); PNec (HR 0.42, 95% CIs 0.30 to 0.61; P < 0.001) and in-hospital mortality (HR 0.51, 95% CIs 0.24 to 1.05; P = 0.036). Moreover, higher median of TG/HDL-C remained significantly associated with POF (HR 1.87, 95% CIs 1.05 to 3.31; P = 0.033), and higher median of T-CHO was statistically related to mortality (HR 0.45, 95% CIs 0.21 to 0.96; P = 0.038).

**Table 4:** Risk of POF, PNec and in-hospital mortality by medians of lipid profiles.

	POF	*P*-value	PNec	*P*-value	In-hospital mortality	*P*-value
Unadjusted HR (95% CI)						
TG (M2 vs. M1)	1.49 (1.09, 2.02)	0.012	2.21 (1.68, 2.90)	**<**0.001	0.98 (0.61, 1.55)	**<**0.001
T-CHO (M2 vs. M1)	0.77 (0.57, 1.05)	0.099	1.27 (0.97, 1.65)	0.077	0.55 (0.34, 0.89)	0.014
HDL-C (M2 vs. M1)	0.20 (0.14, 0.29)	**<**0.001	0.24 (0.18, 0.32)	**<**0.001	0.20 (0.11, 0.37)	**<**0.001
LDL-C (M2 vs. M1)	0.39 (0.28, 0.53)	**<**0.001	0.41 (0.31, 0.54)	**<**0.001	0.33 (0.20, 0.56)	**<**0.001
TG/HDL-C (M2 vs. M1)	2.56 (1.84, 3.55)	**<**0.001	3.22 (2.43, 4.28)	**<**0.001	1.57 (0.97, 2.52)	0.035
Non-HDL-C (M2 vs. M1)	1.08 (0.79, 1.47)	0.634	1.78 (1.36, 2.34)	**<**0.001	0.72 (0.45, 1.16)	0.182
Age- and sex-adjusted HR (95% CI)						
TG (M2 vs. M1)	3.51 (2.37, 5.20)	**<**0.001	2.96 (2.13, 4.09)	**<**0.001	2.96 (1.66, 5.29)	**<**0.001
T-CHO (M2 vs. M1)	0.93 (0.68, 1.28)	0.665	1.32 (1.00, 1.73)	0.057	0.74 (0.45, 1.22)	0.240
HDL-C (M2 vs. M1)	0.17 (0.12, 0.25)	**<**0.001	0.29 (0.21, 0.38)	**<**0.001	0.16 (0.09, 0.30)	**<**0.001
LDL-C (M2 vs. M1)	0.36 (0.26, 0.50)	**<**0.001	0.42 (0.32, 0.55)	**<**0.001	0.29 (0.17, 0.49)	**<**0.001
TG/HDL-C (M2 vs. M1)	6.61 (4.32, 10.11)	**<**0.001	4.20 (3.04, 5.79)	**<**0.001	4.35 (2.45, 7.74)	**<**0.001
Non-HDL-C (M2 vs. M1)	1.37 (0.99, 1.89)	0.062	1.85 (1.40, 2.44)	**<**0.001	1.05 (0.64, 1.74)	0.850
Multivariate-adjusted HR (95% CI)*						
TG (M2 vs. M1)	0.85 (0.47, 1.54)	0.599	0.69 (0.44, 1.10)	0.122	0.58 (0.23, 1.49)	0.258
T-CHO (M2 vs. M1)	0.68 (0.42, 1.09)	0.106	0.98 (0.68, 1.43)	0.929	0.45 (0.21, 0.96)	0.038
HDL-C (M2 vs. M1)	0.28 (0.17, 0.45)	**<**0.001	0.42 (0.30, 0.61)	**<**0.001	0.51 (0.24, 1.05)	0.066
LDL-C (M2 vs. M1)	0.83 (0.54, 1.29)	0.407	0.86 (0.61, 1.22)	0.391	0.79 (0.39, 1.61)	0.520
TG/HDL-C (M2 vs. M1)	1.87 (1.05, 3.31)	0.033	1.06 (0.67, 1.67)	0.806	0.98 (0.41, 2.32)	0.961
Non-HDL-C (M2 vs. M1)	1.09 (0.67, 1.77)	0.723	1.36 (0.92, 1.99)	0.122	0.54 (0.25, 1.15)	0.109

*Multivariate analysis: adjustment for sex, age, biliary etiology, smoking habit, alcohol intake, diabetes mellitus, obesity, history of pre-existing organ dysfunctions including history of chronic pulmonary disease, chronic renal disease and cardiovascular disease, admission laboratory data including total leukocyte count, serum glucose, and calcium.

M1: below the median value, M2: above the median value. HDL-C medians: median 1, <1.06 mmol/L; median 2, ≥1.06 mmol/L; LDL-C medians: median 1, <1.85 mmol/L; median 2, ≥1.85 mmol/L; Non-HDL-C medians: median 1, <3.02 mmol/L; median 2, ≥3.02 mmol/L; T-CHO medians: median 1, <4.14 mmol/L; median 2, ≥4.14 mmol/L; TG medians: median 1, <1.52 mmol/L; median 2, ≥1.52 mmol/L; TG/HDL-C medians: median 1, <1.52; median 2, ≥1.52.

Abbreviations: CI: confident interval; HDL-C, high-density lipoprotein cholesterol; HR, hazard ratio; LDL-C, low-density lipoprotein cholesterol; PNec, pancreatic necrosis; POF, persistent organ failure; T-CHO, total cholesterol; TG, triglycerides.
3. Higher HDL-C quartiles are independently correlated with outcomes

Table 5 shows multivariable-adjusted HRs and 95% CIs for outcomes by quartile of lipid profiles with quartile 1 (lowest quartile) as reference. In contrast to the results for TG, LDL-C, and TG/HDL-C ratio quartiles, there was a negative trend for the association across increasing HDL-C quartiles and incidence of POF, PNec and in-hospital death, p values for trends across quartiles were <0.001, <0.001 and 0.043, respectively. The adjusted HRs for highest HDL-C versus the lowest quartile were 0.20 (95% CIs 0.10 to 0.39) for POF; 0.43 (95% CIs 0.26 to 0.71) for PNec and 0.42 (95% CIs 0.15 to 1.14) for in-hospital mortality, respectively. These data were consistent with, and supported the data, that we obtained for HDL-C and each of the three outcomes.

**Table 5:** HRs for POF, PNec and in-hospital mortality by quartiles of lipid profiles using multivariable analysis.

	1st Quartile	2nd Quartile	3rd Quartile	4th Quartile	*P*-value for trend
POF					
TG	1.00 (reference)	1.61 (0.86, 3.00)	0.95 (0.46, 1.97)	1.99 (0.80, 4.99)	0.352
T-CHO	1.00 (reference)	0.83 (0.45, 1.51)	0.54 (0.29, 1.01)	0.74 (0.38, 1.47)	0.169
HDL-C	1.00 (reference)	0.53 (0.31, 0.92)	0.23 (0.12, 0.43)	0.20 (0.10, 0.39)	<0.001
LDL-C	1.00 (reference)1	0.77 (0.45, 1.34)	0.83 (0.45, 1.53)	0.66 (0.36, 1.20)	0.207
TG/HDL-C	1.00 (reference)	1.67 (0.84, 3.33)	2.02 (0.99, 4.13)	3.68 (1.53, 8.84)	0.005
Non-HDL-C	1.00 (reference)	0.72 (0.39, 1.33)	0.96 (0.52, 1.76)	0.87 (0.43, 1.77)	0858
PNec					
TG	1.00 (reference)	1.83 (1.10, 3.04)	0.75 (0.42, 1.36)	1.95 (0.97, 3.88)	0.314
T-CHO	1.00 (reference)	1.07 (0.64, 1.77)	0.98 (0.60, 1.61)	1.07 (0.61, 1.89)	0.911
HDL-C	1.00 (reference)	0.64 (0.41, 0.99)	0.27 (0.17, 0.45)	0.43 (0.26, 0.71)	<0.001
LDL-C	1.00 (reference)	0.88 (0.56, 1.37)	0.96 (0.59, 1.56)	0.69 (0.42, 1.12)	0.183
TG/HDL-C	1.00 (reference)	1.53 (0.89, 2.61)	0.98 (0.55, 1.75)	2.06 (1.06, 4.02)	0.111
Non-HDL-C	1.00 (reference)	0.76 (0.45, 1.27)	1.28 (0.78, 2.09)	1.04 (0.59, 1.83)	0.474
In-hospital mortality					
TG	1.00 (reference)	2.13 (0.90, 5.05)	1.04 (0.33, 3.23)	0.75 (0.17, 3.36)	0.861
T-CHO	1.00 (reference)	0.39 (0.15, 0.97)	0.23 (0.07, 0.70)	0.42 (0.15, 1.14)	0.017
HDL-C	1.00 (reference)	0.79 (0.34, 1.83)	0.50 (0.19, 1.32)	0.42 (0.15, 1.18)	0.043
LDL-C	1.00 (reference)	0.91 (0.42, 1.96)	0.82 (0.30, 2.23)	0.70 (0.27, 1.82)	0.457
TG/HDL-C	1.00 (reference)	1.78 (0.67, 4.73)	1.67 (0.57, 4.89)	0.71 (0.17, 2.90)	0.942
Non-HDL-C	1.00 (reference)	0.54 (0.23, 1.26)	0.50 (0.20, 1.26)	0.30 (0.10, 0.91)	0.027

*Multivariate analysis: adjustment for sex, age, biliary etiology, smoking habit, alcohol intake, diabetes mellitus, obesity, history of pre-existing organ dysfunctions including history of chronic pulmonary disease, chronic renal disease and cardiovascular disease, admission laboratory data including total leukocyte count, serum glucose, and calcium.

HDL-C quartiles: quartile 1, <0.81 mmol/L; quartile 2, 0.81–1.06 mmol/L; quartile 3, 1.07–1.34 mmol/L; quartile 4, ≥1.35 mmol/L; LDL-C quartiles: quartile 1, <1.26 mmol/L; quartile 2, 1.26–1.84 mmol/L; quartile 3, 1.85–2.36 mmol/L; quartile 4, ≥2.37 mmol/L; Non-HDL-C quartiles: quartile 1, <2.29 mmol/L; quartile 2, 2.29–3.01 mmol/L; quartile 3, 3.02–4.40 mmol/L; quartile 4, ≥4.41 mmol/L; TG quartiles: quartile 1, <0.87 mmol/L; quartile 2, 0.87–1.51 mmol/L; quartile 3, 1.52–4.29 mmol/L; quartile 4, ≥4.30 mmol/L; T-CHO quartiles: quartile 1, <3.36 mmol/L; quartile 2, 3.36–4.12 mmol/L; quartile 3, 4.13–5.45 mmol/L; quartile 4, ≥5.46 mmol/L; TG/HDL-C quartiles: quartile 1, <0.76; quartile 2, 0.76–1.52; quartile 3, 1.52–6.65; quartile 4, ≥6.66.

Abbreviations: CI: confident interval; HDL-C, high-density lipoprotein cholesterol; HR, hazard ratio; LDL-C, low-density lipoprotein cholesterol; PNec, pancreatic necrosis; POF, persistent organ failure; T-CHO, total cholesterol; TG, triglycerides.

Moreover, TG/HDL-C quartile was positively related to POF (adjusted HR for highest quartile was 3.68 [95% CI 1.53 to 8.84]; p value for the trend across quartiles was 0.005). Besides, T-CHO and non-HDL-C concentration quartiles were inversely associated with in-hospital mortality (adjusted HR for highest quartile was 0.42 [95% CI 0.15 to 1.14) and 0.30 [95% CI 0.10 to 0.91], p value for the trend across quartiles was 0.017 and 0.027], with similar but non-significantly different trends for POF and PNec.

We also investigated associations between HDL-C and outcomes in subgroup analyses. The results are shown in Supplementary Tables S1–S3.

### ROC analysis of HDL-C and other lipid profiles as predictors of the three outcomes

**Figure 2:**
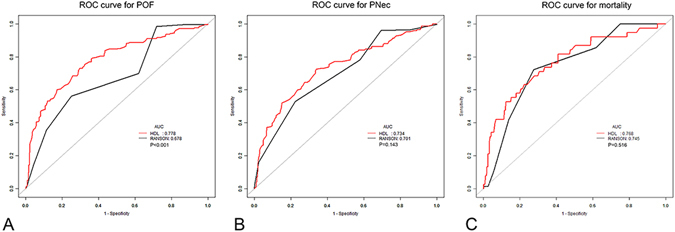
ROC curve of HDL-C levels and Ranson score as pedictors of POF, PNec and in-hospital mortality. Abbreviations: AUC: area under the curve; HDL-C, high-density lipoprotein cholesterol; PNec, pancreatic necrosis; POF, persistent organ failure; ROC, receiver operating characteristic.

We next analyzed the predictive value of these lipid parameters detected upon presentation. Compared to TG, T-CHO, LDL-C, TG/HDL-C and non-HDL-C, HDL-C showed a superior prognostic performance for predicting severe outcomes (Supplementary Table S4). The predictive values of HDL-C for PNec and in-hospital mortality were higher (but not statistically different) than those of Ranson score (AUC 0.734 [95% CIs 0.700 to 0.769] vs. AUC 0.701 [95% CIs 0.668 to 0.734]; P = 0.143 and AUC 0.768 [95% CIs 0.710 to 0.827] vs. AUC 0.745 [95% CIs 0.694 to 0.795]; P = 0.516). Moreover, HDL-C performed significantly better than Ranson score in diagnosing POF (AUC 0.778 [95% CIs 0.740 to 0.85] vs. AUC 0.678 [95% CIs 0.638 to 0.719]; P < 0.001) (Fig. 2). The cut-off value of HDL-C for the prediction of the three outcomes were 0.94 mmol/L (POF), 1.03 mmol/L (PNec) and 0.82 mmol/L (mortality), respectively.

## Discussion

In the present research, we examined the involvement of admission lipid parameters (TG, T-CHO, HDL-C, LDL-C, TG/HDL-C and non-HDL-C) with incidence of POF, PNec and in-hospital mortality in patients with AP. Our results showed for the first time that, in a single-ethnicity Asian Chinese population of patients with AP, even after adjustments for relevant potential confounding factors, a decreased HDL-C level was an independent prognostic factor of adverse outcomes, not only for POF, PNec, but also for death. We also found a decreased LDL-C level was correlated with POF and PNec, but the significance and predictive value were inferior to HDL-C.

AP is an immune disorder featured by the activations of both innate and adaptive immune systems, with release of pro-and anti-inflammatory cytokines. Exaggerated and uncontrollable host response leads to systemic inflammation, and eventually progresses into organ dysfunction and even POF^22^. There exist several pathogeneses contributing to the development of regional pancreatic infarctions that cause PNec, including infiltration of inflammatory cytokines, elevated vascular permeability, hypovolemia with shunting of blood from vital organs, vascular spasm, and free fatty acid (FFA)-induced acinar cell disruption^23, 24^.

HDL plays a central role in FFA clearance and reverse cholesterol transport. Besides, HDL and its mimetics also show anti-oxidant, anti-thrombotic and anti-apoptotic functions^15^. Several researchers figured out that a low amount of HDL-C, which has anti-inflammatory properties, can in turn lead to a more severe systemic inflammatory response^25^. Other laboratories found that HDL can repress inflammatory gene expression in cytokine activated endothelial cells and other cell types^26, 27^.

Apart from the basic function of lipid transference, human LDL is also shown to be involved in organismal protein transfer and delivering pro-inflammatory and pro-thrombotic protein mediators from synthetic place to the site of inflammatory organ systems^28^. In response to inflammatory conditions, native LDL is chemically modified, forming LDL-containing circulating immune complexes (LDL-CIC), which leads to local accumulation and activation of macrophages, releasing pro-inflammatory cytokines and mediators^29^. Modified LDL, especially oxidized LDL, is a key molecule in the early progression of endothelial dysfunction. Studies have demonstrated that ox-LDL plays a central role in the induction of both pro-inflammatory mediators and anti-inflammatory cytokines such as tumor necrosis factor-a (TNF-a), interleukin-6 (IL-6) and interleukin-1-1(IL-1) in human peripheral blood mononuclear cells^30, 31^.

The possible mechanisms causing decreased values of serum lipid cholesterol (including HDL-C and LDL-C) in AP are as follows: the reduction of lipoprotein synthesis in the liver, a lower rate of general catabolic metabolism, and the activation of immune system during the acute phase response. In contrast, the production of triglycerides in the liver increases during the acute inflammatory response^32^.

Decreased values of serum HDL-C in AP represents impaired anti-inflammatory function, which may lead to increase in FFA, creating acidic microenvironment and damaging pancreatic acinar cells. Decreased serum LDL-C is representative of endothelial dysfunction, with elevated vascular permeability, hypovolemia of vital organ systems, and release of both pro- and anti-inflammatory cytokines from immune cells. All of these mechanisms will cause uncontrollable self-immune response and result in the presence of POF, PNec and mortality in AP.

With respect to the relationship between lipoprotein and immune disorders, several studies, including ours, have investigated the association among lipid profiles and outcome in AP. Decreased serum level of HDL-C and decreased activity of paraoxonase 1 (which is responsible for the antioxidant ability of HDL-C) were observed by Unal and colleagues^16^ in an experimental AP model. HDL-C is shown to suppress immune response mediated by toll-like receptor 4 (TLR-4) in macrophages, which is another pathway mediating the anti-inflammatory effects of HDL besides paraoxonase 1^17^. Bugdaci *et al*.^18^ suggested that low levels of HDL in patients with AP during early phase are associated with disease severity. In another study, Khan *et al*.^19^ reported decreased concentrations of serum T-CHO, LDL-C and HDL-C during acute phase in patients with alcohol induced AP compared to normal ones. But the sample size was small and the study did not include other etiologies of AP. Later, they analyzed blood samples obtained within a few days after admission and follow-up samples of patients with AP, and found that serum T-CHO, HDL-C and LDL-C levels measured within 2 days of hospitalization were significantly lower in patients with severe pancreatitis^20^. Peng *et al*. reported serum levels of HDL and Apo A-I at admission can differentiate POF from transient OF in AP, but they failed to include hyper-triglyceridemic pancreatitis^21^.

In spite of AP, low plasma levels of HDL-C is shown as a poor prognostic factor with increased mortality and adverse outcomes in patients with endotoxemia and sepsis^33^. Stachon *et al*.^34, 35^ reported lower serum lipid levels during admission to the intensive care unit in non-survivors compared to survivors. Other researchers reported there was a positive trend for the association across increasing HDL/Apo A-I ratio quartiles and mortality from cardiovascular disease, carcinoma and all cause^36^.

In our previous researches, we found that admission parameters such as peripheral blood CD4+ T lymphocytes^22^, neutrophil to lymphocyte ratio^37^, mean platelet volume^38^, serum calcium^39^ and lactate dehydrogenase^40^ were independent risk factors for POF. The present finding that lower concentrations of admission HDL-C and LDL-C are associated with increased risk of adverse outcomes complements the evidence that decreased HDL-C correlates with acute phase responses. In other words, AP patients presented with low HDL-C and LDL-C levels are at high risk of developing a poor outcome. Moreover, the predictive value of HDL-C is superior to that of LDL-C. Our data suggest that HDL-C levels may have critical influences for other clinical conditions beyond cardiovascular diseases.

Several limitations are evident in our study that need to be considered. First, the observational nature of this study precludes the conclusions of a causal relationship, and as all patients were Asian Chinese, a confounding by ethnic variety cannot be excluded from a theoretical point of view. Second, as a retrospective study, we were not able to measure the concentrations of lipoproteins such as Apo A-I, Apo A-II and Apo B, which may be more specific to HDL-C and LDL-C particles. Third, lipid parameters were measured only once and the time elapsed between assessment and the attack of AP was long in some patients. Moreover, we excluded a large number of patients who do not undergo assessments of lipid profiles upon presentation. This selection bias may influence the results. Besides, we did not include patients with AP induced by trauma as well as patients with chronic pancreatitis. Thus, the conclusion may not be generalized to these kinds of patients. Furthermore, since the baseline values of lipid parameters in AP patients with statin therapy are not within normal range, and statin therapy may influence the outcome in established AP^41–43^, the conclusion may not be generalized to these patients. Due to retrospective nature of our study, we did not record other laboratory parameters beside lipid profiles in these patients. The relationship between lipid profiles and outcomes in AP patients with statin therapy should be explored separately.

There are also several strengths of the present study. First, to the best of our knowledge, this is the first detailed study of the impact of HDL-C on incidence of POF, PNec and in-hospital mortality from a single medical center in an Asian Chinese population of AP. Second, we only include patients who presented within 72 hours from AP symptom onset and without any medical treatment. It is possible that the use of medication such as statin treatment may rapidly influence the levels of peripheral lipid profiles. Furthermore, multivariate adjustments for severity and survival predictors, especially calcium and glucose, were utilized in the outcome analysis.

In conclusion, our results suggest that HDL-C level can independently predict POF, PNec and mortality in patients with AP in an Asian-Chinese population.

## Electronic supplementary material

Supplementary Information
