# Supplementary material for: Decreased high density lipoprotein cholesterol is an independent predictor for persistent organ failure, pancreatic necrosis and mortality in acute pancreatitis
Source: Sci Rep. 2017 Aug 14;7:8064. doi: 10.1038/s41598-017-06618-w (PMC5556036; doi:10.1038/s41598-017-06618-w)
Supplement: Supplementary file 1 — Supplementary Information [file 41598_2017_6618_MOESM1_ESM.pdf]

Title: Decreased high density lipoprotein cholesterol is an independent predictor for persistent organ failure, pancreatic necrosis and mortality in acute pancreatitis

Authors: Yushun Zhang[1*], MD, PhD; Heshui Wu[1*], MD, PhD; Chunyou Wang[1], MD, PhD

Institutional addresses:

[1]Department of Pancreatic Surgery, Union Hospital, Tongji Medical College, Huazhong University of Science and Technology, 1277 Jiefang Avenue, Wuhan 430022, China

To whom correspondence should be addressed:

Yushun Zhang[*], MD, PhD**;** Heshui Wu, MD, PhD[*], Department of Pancreatic Surgery, Union Hospital, Tongji Medical College, Huazhong University of Science and Technology, Wuhan 430022, China.

Email: Yushun Zhang (zhyshun@hust.edu.cn) and Heshui Wu (heshuiwu@hust.edu.cn)

Tel: +86-27-85351621

Fax: +86-27-85351669

Supplementary figure

Supplementary figure 1. Flow chart illustrating patient selection

Abbreviations: AP = acute pancreatitis

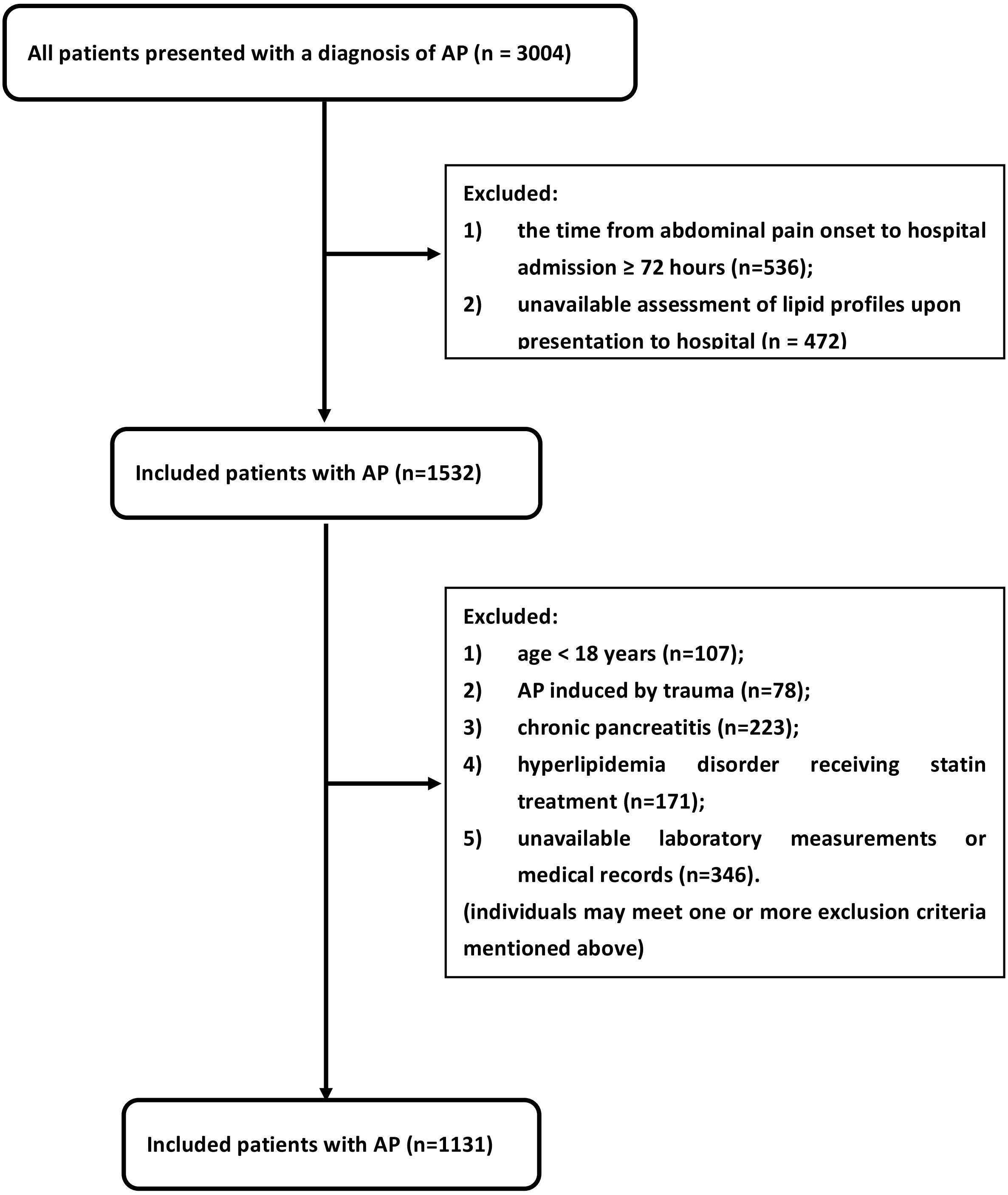

Supplementary tables

Supplementary table 1. Subgroup analysis: incidence of POF according to HDL-C level quartiles

Variables	1st Quartile	2nd Quartile	3rd Quartile	4th Quartile	P for trend	P for interaction
**Subgroup (n)**<br>**HRa (95% CI)**<br>**n (number of POF)**						
**Women (n=537)**<br>**HRa (95% CI)**<br>**n**	1.00 (reference)<br>32	1.32 (0.72, 2.42)<br>30	0.17 (0.07, 0.41)<br>7	0.24 (0.12, 0.48)<br>14	< 0.001	0.762
**Men (n=804)**<br>**HRa (95% CI)**<br>**n**	1.00 (reference)<br>76	0.17 (0.10, 0.29)<br>22	0.11 (0.06, 0.20)<br>14	0.08 (0.04, 0.18)<br>8	< 0.001	
**Age ≥50 (n=542)**<br>**HRa (95% CI)**<br>**n**	1.00 (reference)<br>43	0.44 (0.24, 0.82)<br>32	0.08 (0.04, 0.18)<br>12	0.11 (0.06, 0.23)<br>15	< 0.001	0.001
**Age <50 (n=799)**<br>**HRa (95% CI)**<br>**n**	1.00 (reference)<br>65	0.26 (0.15, 0.45)<br>20	0.13 (0.06, 0.27)<br>9	0.10 (0.04, 0.22)<br>7	< 0.001	
**Daily drinker (n=569)**<br>**HRa (95% CI)**<br>**n**	1.00 (reference)<br>68	0.14 (0.08, 0.26)<br>15	0.16 (0.08, 0.31)<br>11	0.04 (0.01, 0.16)<br>2	< 0.001	0.181
**No daily drinker (n=772)**<br>**HRa (95% CI)**<br>**n**	1.00 (reference)<br>40	0.95 (0.55, 1.64)<br>37	0.12 (0.06, 0.25)<br>10	0.21 (0.12, 0.39)<br>20	< 0.001	
**Current smoker (n=664)**<br>**HRa (95% CI)**<br>**n**	1.00 (reference)<br>70	0.18 (0.10, 0.32)<br>19	0.08 (0.04, 0.18)<br>8	0.04 (0.01, 0.12)<br>3	< 0.001	0.688
**No current smoker (n=677)**<br>**HRa (95% CI)**<br>**n**	1.00 (reference)<br>38	0.83 (0.48, 1.44)<br>33	0.21 (0.11, 0.42)<br>13	0.28 (0.15, 0.51)<br>19	< 0.001	
**Pre-existing co-morbidities (n=410) HRa (95% CI)**<br>**n**	1.00 (reference)<br>40	0.28 (0.14, 0.57)<br>25	0.08 (0.04, 0.18)<br>11	0.17 (0.08, 0.36)<br>15	< 0.001	< 0.001
**No pre-existing co-morbidities (n=410) HRa (95% CI)**	1.00	0.35 (0.21,	0.12 (0.06,	0.07 (0.03,	< 0.001	

**n**	(reference)<br>68	0.58)<br>27	0.24)<br>10	0.17)<br>7		
**Total leukocyte count ≥12×10^9/L (n=616) HRa (95% CI)**<br>**n**	1.00 (reference)<br>61	0.32 (0.17, 0.60)<br>35	0.38 (0.18, 0.82)<br>17	0.17 (0.07, 0.40)<br>12	< 0.001	< 0.001
**Total leukocyte count <12×10^9/L (n=725) HRa (95% CI)**<br>**n**	1.00 (reference)<br>47	0.31 (0.14, 0.68)<br>17	0.08 (0.02, 0.24)<br>2	0.33 (0.13, 0.85)<br>10	< 0.001	
**Serum glucose ≥7.1mmol/L, (n=751) HRa (95% CI)**<br>**n**	1.00 (reference)<br>90	0.32 (0.19, 0.57)<br>40	0.13 (0.07, 0.27)<br>16	0.20 (0.10, 0.42)<br>18	< 0.001	<0.001
**Serum glucose <7.1mmol/L, (n=590) HRa (95% CI)**<br>**n**	1.00 (reference)<br>18	0.41 (0.15, 1.11)<br>12	0.25 (0.08, 0.86)<br>5	0.26 (0.07, 0.95)<br>4	< 0.001	
**Serum calcium <2mmol/L, (n=406)**<br>**HRa (95% CI)**<br>**n**	1.00 (reference)<br>90	0.40 (0.23, 0.71)<br>41	0.19 (0.09, 0.43)<br>13	0.25 (0.10, 0.64)<br>11	< 0.001	< 0.001
**Serum calcium ≥2mmol/L, (n=935)**<br>**HRa (95% CI)**<br>**n**	1.00 (reference)<br>18	0.25 (0.11, 0.60)<br>11	0.15 (0.06, 0.40)<br>8	0.21 (0.09, 0.53)<br>11	< 0.001	

Abbreviations: CI, confident interval; HDL-C, high-density lipoprotein cholesterol; HR, hazard ratio; POF, persistent organ failure. HRa adjustments include sex, age, smoking status, current drinker, pre-existing co-morbidities including history of chronic pulmonary disease, chronic renal disease and cardiovascular disease, total leukocyte count, serum glucose, calcium; but exclude the exposure variable for the individual sub-groups.

Supplementary table 2. Subgroup analysis: incidence of PNec according to HDL-C level quartiles

Variables	1st Quartile	2nd Quartile	3rd Quartile	4th Quartile	P for trend	P for interaction
**Subgroup (n)**<br>**HRa (95% CI)**<br>**n (number of PNec)**						
**Women (n=537)**<br>**HRa (95% CI)**<br>**n**	1.00 (reference)<br>31	1.77 (0.97, 3.24)<br>35	0.20 (0.09, 0.47)<br>8	0.42 (0.23, 0.78)<br>22	< 0.001	< 0.001
**Men (n=804)**<br>**HRa (95% CI)**<br>**n**	1.00 (reference)<br>101	0.27 (0.17, 0.42)<br>52	0.18 (0.11, 0.28)<br>35	0.14 (0.08, 0.25)<br>22	< 0.001	
**Age ≥50 (n=542)**<br>**HRa (95% CI)**<br>**n**	1.00 (reference)<br>37	0.69 (0.38, 1.28)<br>35	0.16 (0.08, 0.32)<br>16	0.29 (0.15, 0.53)<br>25	< 0.001	0.982
**Age <50 (n=799)**<br>**HRa (95% CI)**<br>**n**	1.00 (reference)<br>95	0.45 (0.29, 0.69)<br>52	0.24 (0.14, 0.39)<br>27	0.15 (0.09, 0.27)<br>19	< 0.001	
**Daily drinker (n=569)**<br>**HRa (95% CI)**<br>**n**	1.00 (reference)<br>95	0.24 (0.15, 0.39)<br>41	0.26 (0.15, 0.43)<br>29	0.16 (0.08, 0.31)<br>14	< 0.001	< 0.001
**No daily drinker (n=772)**<br>**HRa (95% CI)**<br>**n**	1.00 (reference)<br>37	1.50 (0.87, 2.56)<br>46	0.19 (0.10, 0.38)<br>14	0.38 (0.22, 0.65)<br>30	< 0.001	
**Current smoker (n=664)**<br>**HRa (95% CI)**<br>**n**	1.00 (reference)<br>94	0.33 (0.21, 0.52)<br>49	0.20 (0.12, 0.33)<br>30	0.14 (0.08, 0.26)<br>19	< 0.001	< 0.001
**No current smoker (n=677)**<br>**HRa (95% CI)**<br>**n**	1.00 (reference)<br>38	1.01 (0.59, 1.74)<br>38	0.21 (0.11, 0.42)<br>13	0.38 (0.21, 0.67)<br>25	< 0.001	
**Pre-existing co-morbidities (n=410) HRa (95% CI)**<br>**n**	1.00 (reference)<br>32	0.56 (0.28, 1.08)<br>28	0.20 (0.09, 0.41)<br>15	0.38 (0.19, 0.79)<br>19	< 0.001	0.008
**No pre-existing co-morbidities (n=410) HRa (95% CI)**<br>**n**	1.00 (reference)	0.50 (0.34, 0.76)	0.20 (0.12, 0.33)	0.16 (0.09, 0.25)	< 0.001	

	100	59	28	25		
**Total leukocyte count ≥12×10^9/L (n=616) HRa (95% CI)**<br>**n**	1.00 (reference)<br>73	0.73 (0.43, 1.22)<br>58	0.61 (0.33, 1.14)<br>29	0.55 (0.29, 1.05)<br>26	0.003	< 0.001
**Total leukocyte count <12×10^9/L (n=725) HRa (95% CI)**<br>**n**	1.00 (reference)<br>59	0.37 (0.19, 0.71)<br>29	0.16 (0.07, 0.34)<br>14	0.32 (0.15, 0.68)<br>18	< 0.001	
**Serum glucose ≥7.1mmol/L, (n=751) HRa (95% CI)**<br>**n**	1.00 (reference)<br>108	0.34 (0.22, 0.54)<br>63	0.14 (0.08, 0.23)<br>29	0.20 (0.12, 0.34)<br>33	0.003	<0.001
**Serum glucose <7.1mmol/L, (n=590) HRa (95% CI)**<br>**n**	1.00 (reference)<br>24	1.02 (0.54, 1.93)<br>24	0.42 (0.21, 0.85)<br>14	0.28 (0.13, 0.59)<br>11	< 0.001	
**Serum calcium <2mmol/L, (n=406)**<br>**HRa (95% CI)**<br>**n**	1.00 (reference)<br>101	0.50 (0.29, 0.87)<br>51	0.44 (0.22, 0.87)<br>24	0.64 (0.28, 1.46)<br>18	0.174	< 0.001
**Serum calcium ≥2mmol/L, (n=935)**<br>**HRa (95% CI)**<br>**n**	1.00 (reference)<br>31	0.67 (0.37, 1.21)<br>36	0.24 (0.12, 0.47)<br>19	0.34 (0.18, 0.64)<br>26	< 0.001	

Abbreviations: CI, confident interval; HDL-C, high-density lipoprotein cholesterol; HR, hazard ratio; PNec, pancreatic necrosis. HRa adjustments include sex, age, smoking status, current drinker, pre-existing co-morbidities including history of chronic pulmonary disease, chronic renal disease and cardiovascular disease, total leukocyte count, serum glucose, calcium; but exclude the exposure variable for the individual sub-groups.

Supplementary table 3. Subgroup analysis: in-hospital mortality according to HDL-C level quartiles

Variables	1st Quartile	2nd Quartile	3rd Quartile	4th Quartile	P for trend	P for interaction
**Subgroup (n)**<br>**HRa (95% CI)**<br>**n (number of in-hospital death)**						
**Women (n=537)**<br>**HRa (95% CI)**<br>**n**	1.00 (reference)<br>16	1.35 (0.63, 2.89)<br>16	-<br>0	0.16 (0.05, 0.48)<br>4	< 0.001	0.207
**Men (n=804)**<br>**HRa (95% CI)**<br>**n**	1.00 (reference)<br>26	0.12 (0.04, 0.36)<br>4	0.27 (0.12, 0.60)<br>8	0.09 (0.02, 0.37)<br>2	< 0.001	
**Age ≥50 (n=542)**<br>**HRa (95% CI)**<br>**n**	1.00 (reference)<br>22	0.63 (0.31, 1.28)<br>18	0.08 (0.03, 0.25)<br>4	0.13 (0.05, 0.34)<br>6	< 0.001	< 0.001
**Age <50 (n=799)**<br>**HRa (95% CI)**<br>**n**	1.00 (reference)<br>20	0.10 (0.02, 0.44)<br>2	0.24 (0.08, 0.71)<br>4	-<br>0	< 0.001	
**Daily drinker (n=569)**<br>**HRa (95% CI)**<br>**n**	1.00 (reference)<br>22	0.08 (0.02, 0.35)<br>2	0.37 (0.14, 0.94)<br>6	-<br>0	< 0.001	0.574
**No daily drinker (n=772)**<br>**HRa (95% CI)**<br>**n**	1.00 (reference)<br>20	0.93 (0.46, 1.86)<br>18	0.06 (0.01, 0.25)<br>2	0.15 (0.06, 0.38)<br>6	< 0.001	
**Current smoker (n=664)**<br>**HRa (95% CI)**<br>**n**	1.00 (reference)<br>20	0.19 (0.06, 0.56)<br>4	0.21 (0.07, 0.64)<br>4	-<br>0	< 0.001	0.019
**No current smoker (n=677)**<br>**HRa (95% CI)**<br>**n**	1.00 (reference)<br>22	0.69 (0.34, 1.39)<br>16	0.13 (0.04, 0.37)<br>4	0.17 (0.06, 0.42)<br>6	< 0.001	
**Pre-existing co-morbidities (n=410) HRa (95% CI)**<br>**n**	1.00 (reference)<br>20	0.48 (0.22, 1.04)<br>14	0.10 (0.03, 0.31)<br>4	0.21 (0.08, 0.57)<br>6	< 0.001	< 0.001
**No pre-existing co-morbidities (n=410) HRa (95% CI)**<br>**n**	1.00 (reference)	0.28 (0.11, 0.71)	0.19 (0.06, 0.56)	-<br>0	< 0.001	

	22	6	4			
**Total leukocyte count ≥12×10^{9}/L (n=616) HRa (95% CI)**<br>**n**	1.00 (reference)<br>22	0.64 (0.26, 1.56)<br>16	0.47 (0.14, 1.52)<br>6	0.32 (0.09, 1.15)<br>4	0.035	0.002
**Total leukocyte count <12×10^{9}/L (n=725) HRa (95% CI)**<br>**n**	1.00 (reference)<br>20	0.23 (0.07, 0.80)<br>4	0.15 (0.03, 0.80)<br>2	0.18 (0.03, 0.96)<br>2	< 0.001	
**Serum glucose ≥7.1mmol/L, (n=751) HRa (95% CI)**<br>**n**	1.00 (reference)<br>36	0.39 (0.17, 0.87)<br>14	0.25 (0.09, 0.72)<br>6	0.19 (0.06, 0.64)<br>4	0.035	< 0.001
**Serum glucose <7.1mmol/L, (n=590) HRa (95% CI)**<br>**n**	1.00 (reference)<br>6	1.09 (0.26, 4.65)<br>6	0.46 (0.07, 3.16)<br>2	0.46 (0.07, 3.09)<br>2	0.075	
**Serum calcium <2mmol/L, (n=406)**<br>**HRa (95% CI)**<br>**n**	1.00 (reference)<br>40	0.39 (0.19, 0.83)<br>16	0.25 (0.08, 0.76)<br>6	0.10 (0.02, 0.48)<br>2	0.025	< 0.001
**Serum calcium ≥2mmol/L, (n=935)**<br>**HRa (95% CI)**<br>**n**	1.00 (reference)<br>2	2.24 (0.26, 19.19)<br>4	0.87 (0.07, 10.62)<br>2	1.50 (0.14, 15.71)<br>4	0.699	

Abbreviations: CI, confident interval; HDL-C, high-density lipoprotein cholesterol;l HR, hazard ratio.

HRa adjustments include sex, age, smoking status, current drinker, pre-existing co-morbidities including history of chronic pulmonary disease, chronic renal disease and cardiovascular disease, total leukocyte count, serum glucose, calcium; but exclude the exposure variable for the individual sub-groups.

Supplementary table S4. Receiving operator curve analyses of lipid files and Ranson score in predicting outcomes

	AUC (95%CI)	Sensitivity	Specificity	PPV	NPV
**POF**					
TG	0.606 (0.562-0.650)	0.394	0.801	0.302	0.852
T-CHO	0.526 (0.472-0.579)	0.261	0.919	0.414	0.850
HDL-C	0.778 (0.740-0.815)	0.729	0.704	0.350	0.922
LDL-C	0.658 (0.615-0.700)	0.557	0.700	0.289	0.878
TG/HDL-C	0.681 (0.639-0.723)	0.581	0.706	0.302	0.885
Non-HDL-C	0.533 (0.472-0.579)	0.305	0.875	0.348	0.852
Ranson score	0.678 (0.638-0.719)	0.562	0.749	0.329	0.887
**PNec**					
TG	0.669 (0.632-0.706)	0.451	0.841	0.513	0.805
T-CHO	0.565 (0.523-0.607)	0.297	0.918	0.572	0.779
HDL-C	0.734 (0.700-0.769)	0.723	0.661	0.442	0.867
LDL-C	0.656 (0.619-0.693)	0.575	0.690	0.408	0.814
TG/HDL-C	0.717 (0.683-0.752)	0.598	0.749	0.469	0.834
Non-HDL-C	0.610 (0.570-0.655)	0.349	0.910	0.584	0.788
Ranson score	0.701 (0.668-0.734)	0.529	0.776	0.467	0.816
**In-hospital mortality**					
TG	0.550 (0.485-0.615)	0.947	0.216	0.080	0.983
T-CHO	0.602 (0.515-0.689)	0.395	0.911	0.242	0.954
HDL-C	0.768 (0.710-0.827)	0.632	0.777	0.170	0.967
LDL-C	0.678 (0.609-0.747)	0.658	0.652	0.120	0.964
TG/HDL-C	0.630 (0.567-0.694)	0.290	0.907	0.183	0.947
Non-HDL-C	0.562 (0.477-0.647)	0.263	0.963	0.339	0.948
Ranson score	0.745 (0.694-0.795)	0.724	0.723	0.158	0.973

Abbreviations: AUC: area under the curve; CI: confidence intervals; HDL-C, high-density lipoprotein cholesterol; HR, hazard ratio; LDL-C, low-density lipoprotein cholesterol; NPV: negative predictive value;PNec, pancreatic necrosis; POF, persistent organ failure; PPV: positive predictive value; ROC, receiver operating curve; T-CHO, total cholesterol; TG, triglycerides.
